# DAILY—A Personalized Circadian *Zeitgeber* Therapy as an Adjunctive Treatment for Alcohol Use Disorder Patients: Study Protocol for a Randomized Controlled Trial

**DOI:** 10.3389/fpsyt.2020.569864

**Published:** 2021-01-14

**Authors:** Anisja Hühne, Eva Hoch, Dominic Landgraf

**Affiliations:** ^1^Circadian Biology Group, Department of Molecular Neurobiology, Clinic of Psychiatry and Psychotherapy, University Hospital, Ludwig Maximilian University, Munich, Germany; ^2^Munich Medical Research School, Ludwig Maximilian University, Munich, Germany; ^3^Cannabinoid Research and Treatment Group, Division of Clinical Psychology and Psychological Treatment, Department of Psychology, Clinic of Psychiatry and Psychotherapy, University Hospital, Ludwig Maximilian University, Munich, Germany

**Keywords:** circadian, alcohol use disorder (AUD), *Zeitgeber*, daily structure, addiction, chronotherapy, depression, personalized therapeutic approach

## Abstract

**Background:** Hallmarks of alcohol use disorder (AUD) are disturbances of circadian rhythms and everyday structures. While circadian rhythms dictate the timing of daily recurring activities such as sleep, activity, and meals, conversely, these activities represent time cues, so called *Zeitgebers*, that the circadian system uses to synchronize with the environment. Here we present a study protocol for our newly developed therapy approach for AUD patients, in which we take advantage of this mutual influence and stabilize and strengthen their circadian system by creating strict daily schedules for daily *Zeitgeber* activities. Since every person has a circadian system with its own characteristics and is subject to social obligations, the daily plans are personalized for each test person. Our hypothesis is that a regular exposure to *Zeitgebers* stabilizes behavioral and physiological circadian rhythms and thereby reduces the risk of alcohol relapses and depressive symptoms and facilitates physical recovery in AUD patients during the 1st weeks of their addiction therapy.

**Methods/design:** The study is a 6-weeks single site trial with a controlled, randomized, single-blinded, parallel-group design including patients with a diagnosis of AUD. The study runs parallel to the standard addiction therapy of the clinic. Patients are randomly assigned to either an intervention group (DAILY) or a sham control group (placebo treatment). Questionnaires and physiological assessments of both groups are conducted before and immediately after the intervention or control treatment. According to our hypothesis, the primary outcomes of this study are improvements of regularity, alcohol consumption, and relapse rate in AUD patients compared to AUD patients receiving control treatment. Secondary outcomes are reduced depressive symptoms and increased physical recovery.

**Discussion:** This study is a randomized controlled trial to investigate the efficacy of a personalized circadian *Zeitgeber* therapy as an adjunctive treatment for alcohol use disorder patients. The overall goal of this and more extended future studies is the development of an adjunctive therapy for AUD patients that is uncomplicated in its use and easy to implement in the clinical and everyday routine.

**Trial registration:** This study is registered at the German Clinical Trial Register with the trial number DRKS00019093 on November 28, 2019.

## Background

### Harmful Use of Alcohol and Alcohol Use Disorder

Harmful use of alcohol and alcohol use disorder (AUD) lead to increased morbidity and mortality. According to the World Health Organization (WHO), 5.1% of all disorders, diseases, injuries, and 5.3% of all deaths are attributable to alcohol consumption (1). Typical disorders and diseases associated with harmful alcohol consumption and AUD include cardiovascular, infectious, neurological diseases, cancer, and psychological disorders such as depression and anxiety (2, 3). Impressively, as a result, mortality due to alcohol consumption is higher than for other serious diseases such as HIV or tuberculosis (1). In addition to the adverse health effects of those concerned, this also means that, at the societal level, harmful alcohol consumption behavior creates a significant burden on the economy and health systems (4, 5).

### The Circadian System

Endogenous circadian clocks (from Latin *circa diem*, about a day) have evolved to prepare organisms to daily recurring environmental changes caused by the rotation of the earth around its axis. Circadian clocks are found in almost all cells of the body and since they regulate the expression of about 50% of our genes (6), there is almost no process in the body that is not under their influence. In humans, the master circadian clock is located in the suprachiasmatic nucleus (SCN) in the anterior part of the hypothalamus (7–9). The SCN acts like a conductor of an orchestra and sets all other cellular clocks in the rest of the body to an appropriate period and phase (10). Synchronization of endogenous circadian rhythms with environmental 24-h cycles is achieved through so called *Zeitgebers* (German: time givers) (11). The strongest stimuli that serve as *Zeitgebers* are light and food, whereby light acts directly on the SCN. In a simplified model, the SCN, in turn, determines sleep and wake times and when hunger arises. The ingested food then entrains peripheral clocks. Physical activity (exercise) and social contact are also regarded as *Zeitgebers*, but weaker ones (12). In an optimal situation, molecular, physiological, and behavioral rhythms are synchronized among each other and with the environment (13). Since the circadian clock is involved in virtually all bodily processes, their perturbation is strongly associated with a high number of disorders, including mental illnesses such as AUD or major depressive disorder (MDD) (13, 14).

### Association of Circadian Clocks and Alcoholism

In recent years evidence emerged that there is a bidirectional relationship between circadian rhythms and alcohol consumption (15). AUD patients often suffer from circadian misalignment, which, in turn, is known to increase the risk of developing AUD (15–17).

On the one hand, individuals with AUD almost always suffer from disorders of the circadian system, such as disturbed sleep times, impaired sleep quality, altered molecular rhythms, and altered daily rhythms of neuroendocrinological functions in different regions of the brain (18). Even acute alcohol drinking can cause spontaneous alterations in circadian rhythms (19), and circadian rhythms are also affected during alcohol withdrawal (20, 21). Importantly, alcohol-dependent patients have a higher risk of relapse during withdrawal if their internal clock is disturbed (18). On the other hand, disrupted circadian rhythms are considered a strong risk factor for the development of AUD, as disturbances of circadian sleep-wake cycles are frequently observed in subjects with a late chronotype and shift workers show an increased prevalence of harmful use of alcohol and AUD (19). Moreover, single nucleotide polymorphisms in the clock genes Clock, Per2, and Per3 genes have been associated with alcohol consumption behavior (22, 23).

Curiously, although AUD patients generally suffer disturbances of molecular and behavioral rhythms, their need for alcohol often follows circadian patterns with around 80% of alcohol addicts having their first drink at a specific hour with low variability (17, 24–27). Accordingly, in rodents, the regular administration of drugs can trigger anticipatory locomotor activity before the drug is actually provided, which suggests the existence of a drug-entrainable oscillator (24).

In summary, AUD patients have disturbed circadian rhythms in daily sleep and neuroendocrinological rhythms on the one hand, but on the other hand are strongly entrained by alcohol. Together, these data highly suggest an imbalance of *Zeitgeber* signals in AUD patients. While light, food, and physical activity are usually the strongest *Zeitgebers*, in AUD patients these may lose influence and be replaced by alcohol. Thus, a targeted amplification of the other *Zeitgebers* can possibly reduce the influence of the *Zeitgeber* alcohol and thereby reduce symptoms of AUD patients during withdrawal and help to reduce relapses.

### Biological Mechanisms Targeted With a Circadian *Zeitgeber* Therapy in AUD Patients

In recent years, there has been growing evidence that circadian rhythms modulate reward circuits and behavior (28–31). For instance, the activation of reward processing is subject to circadian control (32), the release of neurotransmitters, such as dopamine and serotonin, varies over the course of the day, and dopamine signaling in the SCN has strong impact on its rhythms and processing responses to hedonic stimuli (33, 34). Thus, in animals and humans, depending on the time of day, rewards are valued differently (35) with the consequence that sensitization and motivation toward rewarding stimuli change over the course of 24 h (32, 36–38). Moreover, in humans, under constant conditions, it was shown that rhythms of the diurnal positive affect parallels rhythms of core body temperature, further suggesting endogenous circadian control of reward motivation (39).

Because of the close connection to the reward system, it is not surprising that circadian clocks have also been associated with addiction and alcohol use (16, 40, 41). Circadian misalignment in humans can be proven and quantified by the discrepancy between the phase angles of the dim light melatonin onset (DLMO) and the sleep offset. Interestingly, the more pronounced the circadian misalignment is, the greater the severity of substance abuse and dependence (42). It is also remarkable that melatonin levels, which are usually high at night, are inverted in AUD patients under the influence of alcohol and during acute withdrawal, which further indicates severe circadian disturbances in AUD patients (43). Moreover, circadian misalignment due to a late chronotype is also associated with increased impulsivity, which is a risk factor for the development of AUD (44, 45). Furthermore, it was shown that at certain times of the day, craving is particularly pronounced, since our reward system follows a circadian pattern (27).

In rodents, drug reward displays daily rhythms (46). These rhythms are associated with oscillating expression of the dopamine transporter and are directly related to clock gene expression in the medial prefrontal cortex and mesolimbic dopaminergic system (47). Mice exposed to chronic circadian disruption display reduced neuronal complexity in prelimbic prefrontal brain areas and reduced behavioral inhibition, which again is associated with increased alcohol consumption (48).

The above described studies indicate the existence of a neurobiological circadian-reward system, which in case of disruption can contribute to the development and maintenance of addiction disorders (40). Therefore, our therapeutic approach aims to synchronize circadian rhythms of the entire body among themselves and with the environment by a very regular exposure to *Zeitgebers* (light, dark, food, and if applicable activity). In a stable light-dark cycle, the SCN entrains robustly to it, and can reliably transmit these time signals to the rest of the body. Additionally, food is a very potent *Zeitgeber*, and regular mealtimes can entrain clocks of most peripheral organs as well as many brain oscillators (49, 50). Among those are brain areas that are involved in motivational responses to food and the SCN (51, 52). Consequently, strictly timed food has already been suggested as chronotherapeutic approach to improve circadian disruptions in populations such as shift workers (53, 54).

### Circadian *Zeitgeber* Therapy for AUD Patients in the Transition From Inpatient to Outpatient Care

For AUD patients, the transition phase between the inpatient stay and discharge from the clinic to outpatient care or home often represents a stressful and thus susceptible time for alcohol relapses and a worsening of the general psychiatric health situation (55). For patients the sudden change from the clinical environment back to autonomous self-organized daily routines is often very difficult (56). During this transition phase, daily rhythms such as sleep-wake cycles and mealtimes become irregular and often drift into a later phase, which has been associated with a worsening of psychiatric symptoms, including alcohol relapses and depression (57, 58).

Within the framework of psychotherapy the suggestion of structuring the everyday life in an orderly manner to psychiatric patients, especially AUD patients is not new (59). This therapy component of self-management has positive psychological effects such as reducing stressful situations and enhancing the feeling of self-efficacy. However, we have designed a study that, in addition to previous therapy programs, has a special focus on the transition time between inpatient and outpatient care or home, and which focuses on the targeted use of strong *Zeitgebers* to restore and stabilize physiological circadian rhythms in AUD patients. Similar circadian reinforcement therapies have been proven for bipolar disorder (60, 61) and are currently tested in a study for MDD (62). However, the development and systematic investigation of a circadian structure therapy for AUD patients has not yet been published and was recently highlighted as a necessity (63). Thus, with DAILY we develop a new treatment approach for AUD patients, partly based on established concepts of chronotherapies for other mental disorders and implement it alongside standard AUD therapies.

We assume that subjects in our study benefit from the reduction of physical conditions that promote and maintain alcohol consumption behavior. We further believe that the strengthening of circadian rhythms has not only positive effects on alcohol craving, but also on often accompanying symptoms of AUD, such as depressive mood. Therefore, we have called this newly proposed intervention for AUD patients DAILY, which stands for **D**epression **A**lcohol **Il**lness Therap**y**.

## Study Hypotheses and Objectives

Our primary hypothesis is that a regular exposure to *Zeitgebers* stabilizes circadian behavioral rhythms and thereby reduces alcohol consumption and the risk of alcohol relapses in AUD patients. Accordingly, the primary objectives of the study are to determine whether the intervention program DAILY decreases day-to-day variations in behavior and reduces alcohol consumption and the relapse rate in AUD patients compared to those receiving control treatment. Furthermore, we hypothesize that the regular exposure to *Zeitgebers* contributes to the reduction of depressive symptoms and a subjective and physiological well-being in AUD patients, which in turn positively affects the primary objectives of the study. Accordingly, the secondary objective of the study is to ascertain whether the DAILY program supports the surrogate criterions of increased sleep quality, reduced depressive symptoms, increased self-efficacy expectations, and improved blood-values that are associated with harmful alcohol consumption. We also hypothesize, that additional psychoeducation about biological mechanisms in the development of AUD helps participants to complete their current therapy and maintain compliance. Therefore, as exploratory outcomes, we will investigate whether more participants of the intervention group accomplish the DAILY therapy than participants of the control group. Finally, we will assess whether the DAILY intervention has impact on the chronotype of the participants, although it is not a specific aim of our treatment to change it.

## Study Design

### Design

The study is a 6-weeks trial with a controlled, randomized, single-blinded, parallel-group design including patients with a diagnosis of AUD. Patients are randomly assigned to either an intervention group (DAILY) or a sham control group (placebo treatment). As this study represents a pilot randomized trial, which does not primarily focus on proving treatment successes but more on ascertaining the best options for the future main trial, we will include each eligible patient within this year instead of using formal power calculations (64).

### Setting

DAILY is a single site study conducted at the specialist ward for addiction disorders and the outpatient clinic for substance use disorder of the Clinic for Psychiatry and Psychotherapy at the University Hospital of Munich. There are 24 patients with a minimum age of 18 years on the ward, with men and women admitted equally. The day clinic treats 15 patients with a minimum age of 18 years simultaneously. On the ward and in the day clinic there are fixed, individually determined schedules for therapy sessions from which patients are not allowed to deviate. Meals are offered by the clinic at fixed times of the day, but do not have to be taken. Patients are free to leave the ward and the day clinic between therapy sessions in order to take meals outside or to pursue other activities such as walks or sports. The ward is open in principle, but care is taken to ensure that patients do not leave it between 10 pm and 8 am. There are no fixed sleeping hours on the ward, except that patients must arrive on time for their therapy sessions, which usually start between 08:30 and 11:00 am. On the weekend, patients of the ward have no regular appointments. Patients of the Day Clinic leave the clinic in the afternoon and arrange the rest of the day and their weekends independently. In addition, food can be brought to the ward and the day clinic and snacks can be eaten at any time. Both clinics are interdisciplinary staffed by medical doctors, psychologists, nurses, and social workers, all of whom have broad experience in the treatment of substance use disorders, including AUD. About half of the patients on both sites are diagnosed with AUD, the other half suffers from multidrug dependency. On the ward a qualitative detoxification program with psychotherapeutic services is offered to AUD patients and patients with multidrug use. In the day clinic the same psychotherapeutic services are offered as on the ward, but the patients are already detoxified, and the therapy takes place on a day-care basis.

### Standard Therapeutic Services on the Ward and the Day Clinic

In parallel to the DAILY or the sham treatment, all patients receive the standard clinical treatment consisting of medical and psychological consultation, individual and group therapy (combining Motivational Enhancement Therapy and Cognitive Behavioral Therapy), and medication if necessary. The psychotherapeutic treatment of AUD and multiple substance use patients is the same but is conducted separately. The standard psychotherapy concept of the ward and the day clinic includes motivation work in which the incentive to abstinence and the decision to change consumption behavior is increased. In addition, situation analyses, the identification of risk factors, and the development of alternative actions are used to work with patients on relapse prevention. In addition, all patients receive psychoeducational therapy sessions to provide theoretical knowledge about the definition of addiction and its development on a neurobiological level. The treatment of AUD patients on the ward is scheduled for 14 days. In the day clinic, the treatment time ranges from 2 to 4 weeks and the concepts described above are elaborated in more detail and sociotherapy is also offered. In none of these standard therapies the advantages of daytime structures and regular sleep times are discussed.

### Study Population

Men and women, unemployed and employed subjects are equally included in the study. Depending on whether the subjects are patients of the ward or day clinic, they are in detoxification or post-detoxification treatment. Study enrollment of subjects who are newly admitted to the detoxification program of the ward will take place at the earliest after 5 days of detoxification, to exclude cognitive and physical limitations caused by acute intoxication and its after-effects. Study participants who are discharged from the ward either go home, receive further treatment in the day clinic, or go to other institutions for long-term therapy. Study participants who are discharged from the day clinic either go home after discharge or go to other institutions for further treatment. The health conditions of all test persons allow them to perform their daily tasks without external assistance. Approximately half of the study subjects have a regular job, while the others are unemployed or retired. Usually 3–5 subjects are involved in the study simultaneously.

### Eligibility Criteria

All persons are considered eligible on whom the below exclusion criteria do not apply. Subjects are only included in the study if they can assess the nature and scope of the investigations. Written consent is required for inclusion. The study is conducted in accordance with the principles of the Declaration of Helsinki with its amendments of Tokyo, 1975, Hong Kong, 1989, Somerset West, 1996, Seoul, 2008, and Fortaleza, 2013. The subjects must fulfill diagnostic criteria for AUD according to ICD-10 F10.2 and be of age 18–75 years. Smokers are eligible for this study. In case of dependency on other substances, the subjects must have been abstinent from these substances for at least 12 months at the time of study enrollment. One outcome of this study is the change in depressive symptoms in the subjects. For this reason, AUD patients with concomitant diagnosed MDD as well as substance-induced depression are included. However, a diagnosed depression is not a prerequisite for inclusion in the study.

### Exclusion Criteria

Excluded are subjects who are unable to give their consent, are pregnant, or are currently working in shifts. In addition, intellectual, neurological, or physical impairment that lead to the inability to independently perform tasks of daily life lead to exclusion of the study. In general, dependence on help with eating or going to bed (e.g., bedridden patients) precludes participation in the study. However, patients with disorders like liver damage, tremors, digestive problems, and blood abnormalities, which are common in AUD patients but do not lead to constant dependence on support from others, are not excluded. Blind subjects and patients with diagnosed mental illnesses other than AUD and MDD are generally excluded from the study. Patients with a dependence on other substances (except alcohol and nicotine) which they have consumed within the last 12 months are also not ineligible. The use of benzodiazepines, agomelatine, or medically prescribed cannabinoids will result in exclusion from the study, the use of other medications will not affect eligibility. Although patients with depressive symptoms are generally eligible for the study, no psychotic symptoms or acute suicidal ideation should be present. Subjects must not participate in other studies that may interfere therapeutically with the DAILY study.

## Study Procedure

**Figure 1:**
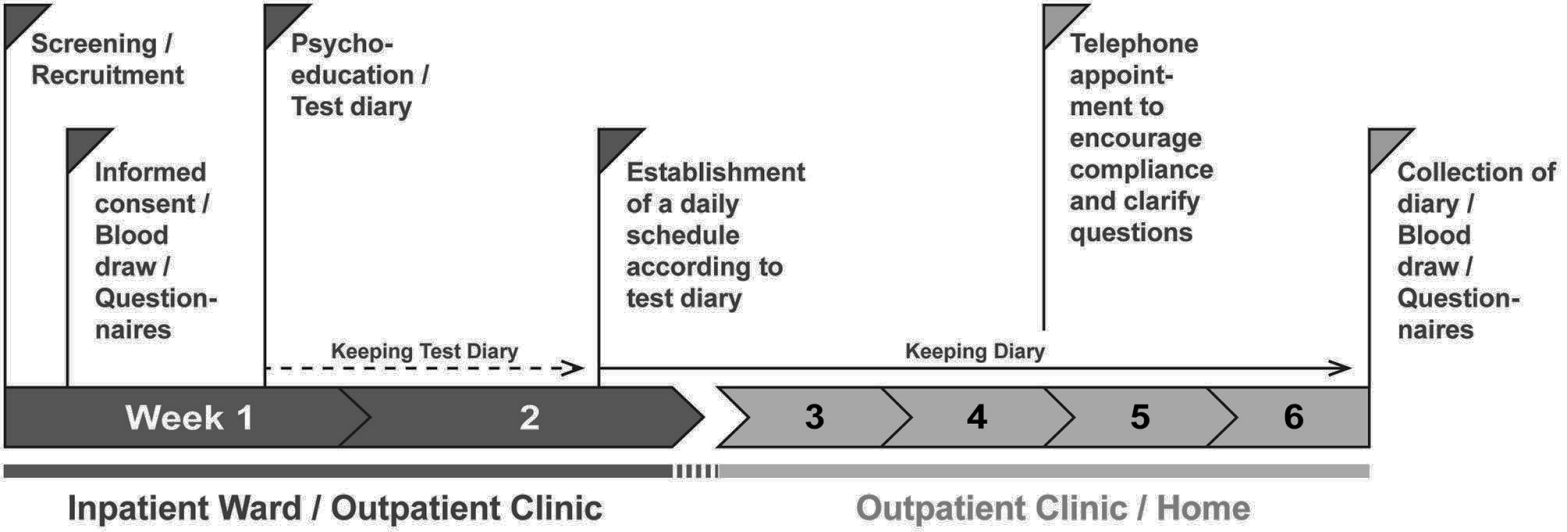
Procedure of the DAILY study. Patients are screened and recruited by a member of the study staff. After 1 day at the earliest the participants sign the consent form. On this day, they are informed about the contents of the study; the contents discussed differ between intervention and control group. In addition, questionnaires are filled out and blood is drawn. During the week, a psychoeducation session takes place in which the contents and the aims of the study are deepened, further questionnaires (Table 1) are filled in, and a test diary is handed out to record times of daily actions which act as *Zeitgebers*. After at least 1 week, the diary is evaluated and a daily structure plan for regular exposure to *Zeitgebers* is worked out together with the test persons, to which they should adhere for the next 4 weeks. Subjects in the control group are not given instructions on how to structure their daily routine but are still asked to complete the diary. At least one telephone appointment is made for the time the subjects fill out the diaries to maintain compliance. After 4 weeks, there will be a final personal appointment where the diaries will be collected, all questionnaires will be repeated, and blood will be drawn again.

The study procedure for the study participants is shown in Figure 1. The basic procedure is the same for participants of the intervention and control group.

### Recruitment

**Figure 2:**
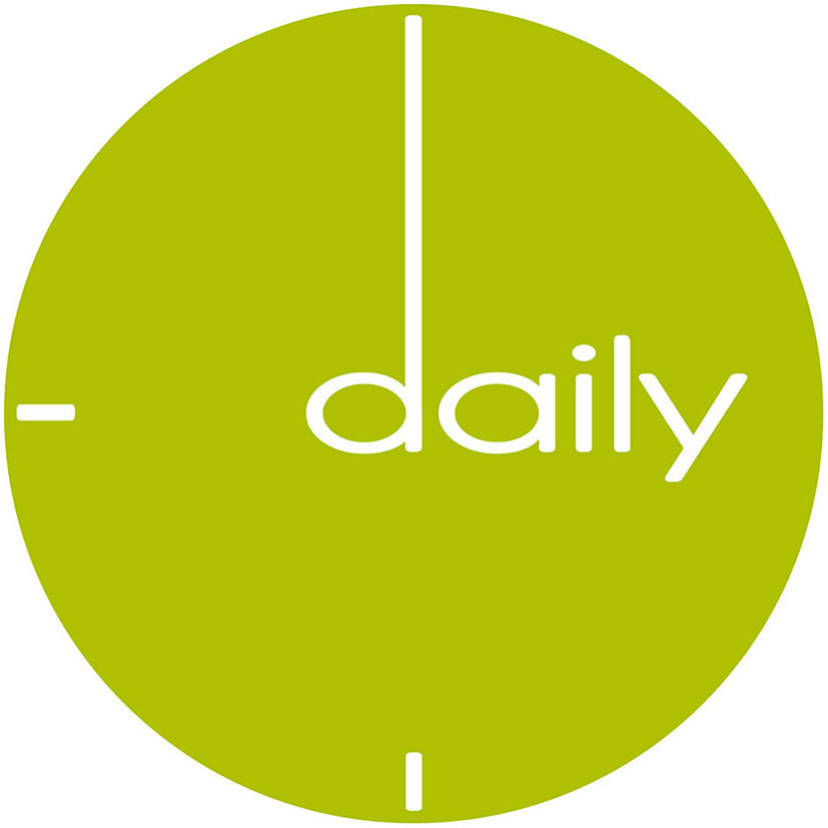
Logo of the DAILY therapy study. The logo represents a clock in which the letters act as hands. The clock is set at a full hour, which should underline the desired regularity in everyday life. The color green is synonymous with life and health.

In order to facilitate recruitment, a logo was designed for the DAILY study, which has a high recognition value for the test persons and underlines the official character of the study (Figure 2). After screening for eligibility, participants are recruited during their clinical stay at either the specialist ward or the outpatient clinic for addiction disorders. A member of the study staff addresses the candidates personally and finds out whether there is in principle interest in participating in a concomitant therapy study for AUD patients. Candidates are given at least 1 day to consider. In the event of positive feedback, they are allocated to either the intervention or control group.

### Allocation

Participants are allocated to either the intervention or control group by a computer-generated randomization list. Each test person is assigned an individual study code, which contains information about the current ward and gender, and otherwise consists of a sequential number. Personal data that could reveal identity and participation in the intervention or control group are not part of the code. The decoding of the code is only allowed for study staff. Until the end of the study, the participants will not be informed about their assignment to the intervention or control group. However, after the end of the study, participants will be informed about their group affiliation and participants of the control group will be given the opportunity to learn about the circadian system and the importance of a structured daily routine.

### Informed Consent and Baseline Assessments

To participate in the study, all participants need to sign an informed consent in the 1st week (Figure 1). Afterwards, irrespective of the group allocation, a semi-structured interview is conducted to assess demographical data: age, gender, employment status, occupation, and marital status. Medical data are evaluated as well: size, bodyweight, blood pressure, current medications, and information about previous alcohol problems and possible depressive episodes. In addition, the questionnaires described below (Table 1) are filled out together with the participants. Lastly, blood samples are collected. This appointment is also used to prepare the test persons for the topics and contents of their respective groups.

**Table 1:** Overview of questionnaires, physiological assessments, and time points.

**Assessment category**	**Assessment instruments**	**First session**	**Last session**	**Between sessions**
Baseline	Demographics	x		
Circadian	Sleep-Food-Diary			x
	Munich ChronoType Questionnaire (MCTQ)	x	x	
	Pittsburgh Sleep Quality Index (PSQI)	x	x	
Alcohol use	Alcohol use disorders identification test (AUDIT)	x	x	
	European Addiction Severity Index (EuropASI)	x	x	
	Timeline Followback 30 (TLFB)	x	x	
Depression	Hamilton Depression Scale (HAMD)	x	x	
	Inventory of Depressive Symptomatology (IDS-SR)	x	x	
Self-efficacy	Self-Efficacy Questionnaire (SWE)	x	x	
Blood values	Blood draws	x	x	

The further course of the study and the contents of the following appointments are described separately for the intervention group and the control group.

### Intervention Group

#### Week 1: Psychoeducation and Test Diary

After baseline assessments, a few days later in the same week, the test persons participate in individual 1-h psychoeducation sessions about circadian clocks and their effects on health and psychiatric disorders, such as addiction and depression. The scientific content is communicated in simple language. Firstly, the participants get explained that circadian rhythms can be found virtually everywhere in nature and that they have impact on essentially all physiological processes, including sleep-wake cycles, hunger, digestion, wound healing, cellular functions, and molecular processes. A focus is on their involvement in neurobiological systems, such as reward, addiction networks, and behaviors. During the session, the test subjects are led to understand how important regular daily routines are for mental and physical health. Therefore, the study assistant emphasizes the importance of certain environmental cues that can serve as *Zeitgebers*, such as light and food. Together with the participants, the impact of light on sleep is discussed and how light can be avoided at late hours (e.g., by stopping the use of mobile phones, watching TV, or playing computer games after sunset or by using blue-light filters). Furthermore, the importance of regular mealtimes is discussed as food is an important *Zeitgeber* for many organs, including brain areas involved in the development of addiction. At the end of this session, the study assistant hands out a circadian diary (Supplementary Table 1), which the participant is asked to fill out for 1 week. With the help of the diary, data on times of meals, snacks, activity times, sleep duration, sleep quality, mood, and alcohol craving are collected on a daily basis. At this point in time, the test persons do not yet receive any concrete instructions as to when they should execute activities. Rather, they are instructed to use the following days to see if they can recognize certain temporal patterns in their own behavior and to consciously follow these patterns. Filling out the test diary serves on the one hand to create an awareness of their own daily rhythms. Besides, it gives the test persons the opportunity to familiarize themselves with the filling out of the diary. On the other hand, the data obtained on eating and sleeping times can be used in the next session as a basis for drafting a daily structure plan.

#### Week 2: Establishment of a Daily Structure Plan

After 1 week, participants are invited for another individual session. During this session, the entries of the test diary are discussed and a personalized daily structure plan for eating and sleeping times and, if desired by the test subjects, times for leisure activities such as sports is created together with the participants. For each subject, a balance is attempted between endogenous circadian preferences and time constraints imposed by the environment, which the subjects may not be able to influence. Thus, it is sought to adapt all possible freely selectable times to the personal preferences given by individual circadian characteristics. However, some test persons are embedded in certain rigid daily structures on some days (e.g., due to working hours). These restrictive environmental factors are also taken into account when creating the daily structure plan in order to ensure their realistic implementation in everyday life and to avoid possible sleep deprivation due to late bed and early work hours. If this is the case, it is recommended to the test persons to strictly adhere to the daily schedule of working days even on non-working days in order to avoid repeated shifts in the circadian system and to maintain a maximum of regularity, and therefore endogenous synchronization. However, for each individual subject, it must be checked whether the daily schedule created for them deviates significantly from their endogenous circadian preferences and thus leads to a permanent misalignment between endogenous and exogenous rhythms or sleep deprivation. If the discrepancy is larger than 2 h, a specific adjustment of the lighting conditions is recommended, e.g., for late chronotypes with early work hours, increasing the light in the morning (bright light therapy) to advance the circadian system and to promote wakefulness, and decreasing it in the evening to further support the phase advance and promote tiredness.

Furthermore, at this meeting the test persons are again made aware of the importance of very regular eating and sleeping times. If the test diary shows that the test person has lived rather irregularly, solutions are developed for maintaining more daily regularity. This session also serves as an opportunity to answer potential questions from the study participants and to remind them of the importance of adhering to the daily structure plan that has been drawn up. Participants receive new blank diaries which they are asked to fill in for the next 4 weeks. The times of the designed daily structure plan are handwritten in the empty diary pages as a visual support for precise adherence to the times.

#### Weeks 3–6: Self-Employed Application of the Daily Structure Plans, Telephone Appointment, and Final Assessment

Starting from this point in the study, the test persons are asked to independently implement the previously developed daily plan for the next 4 weeks and to continue filling out their diaries. The implementation of the daily structure plan can already start at times when the patients are still on the ward or in the day clinic, as patients are allowed to choose the times of all activities (going to bed, eating, leisure activities) freely, with the exception of the fixed therapy sessions on weekdays (see above). However, many patients are discharged or transferred to another institution at different times during this phase of the study. Patients who are discharged home will be asked to follow up the daily schedule outside the clinical setting. Patients who continue their treatment in other institutions will be asked to maintain their daily schedule as much as possible in their new environment. During this time, they can contact the study staff for further assistance (i.e., to adjust the daily structure plan). After 2 weeks, participants are contacted by the study staff by telephone. The telephone call follows the rules of motivational interviewing (65) in order to motivate and remind the participants to further keep to the agreed daily structure and to continue to fill in the diaries thoroughly. If desired, patients are asked to send already completed diaries by fax, mail, or email. The continuous completion of the diary during the application phase of the study serves not only for continuous data collection but also for self-monitoring and increasing compliance. Another 2 weeks later, a last individual appointment is arranged. During this session, diaries will be collected, the questionnaires from the first session are repeated and another blood sample is taken (Table 1).

### Control Group

The control group follows the same conceptual procedure and sequence as the intervention group (Figure 1). Thus, they have the same number of individual sessions and accordingly receive same attention from the study staff. They also fill out all the questionnaires, give blood, and fill out the diary for the same amount of time. However, there are differences in the content of the sessions, in that the control group will not receive information about the circadian clock, its connection to addiction, and the importance of having a structured daily life. Instead, participants of the control group are given the opportunity to discuss, for example, the role of legal drugs, such as alcohol, in society and the effects of alcohol advertising and its impact on both individual and societal consumption behavior. Since the test persons of the control group do not receive any further instructions for the diary and are only asked to fill it in carefully, the evaluation of the study data provides the possibility to compare Routine Variability Scores of test persons of the intervention group and the control group.

## Measured Outcomes

Our primary hypothesis is that a regular exposure to *Zeitgebers* stabilizes circadian behavioral rhythms and thereby reduces the risk of alcohol relapses in AUD patients. Therefore, the first primary outcomes of the study are the Routine Variability Score and drinking behavior of each individual test person. For generation of the Routine Variability Scores, first, day-to-day variability will be calculated from the following parameters: bedtime, wake-up time, sleep quality, time of getting-up in the morning, time to go to bed in the evening, lights-off time, breakfast time, lunch time and dinner time. The individual Routine Variability Score is comparable to the validated social rhythm metric scale (SRMS) (66) and will be calculated as follows (example for bedtime): For the period of 1 week, the average bedtime will be calculated. Then the deviation from this average bedtime is calculated in minutes for each day. These deviations are summed up and the mean value is calculated. This mean value will be coded according to the magnitude of time variability: 1: 0–15 min, 2: 16–30 min, 3: 31–45 min, 4: 46–60 min, 5: 61–75 min, 6: 76–90 min, 7: 91–105 min, 8: 106–120 min, 9: 2–3 h, 10: 3–4 h, 11: over 4 h (67). This way a separate score can be created for each parameter collected in the diary. These scores can be calculated on a weekly basis to examine whether relapses or deterioration in well-being are related to irregularities in the daily routine during that week, or over the duration of the entire survey phase to check how regularity over a longer period is related to drinking behavior and well-being. In addition, the scores for sleep and meals, for example, can be calculated individually to examine their influence on drinking behavior and well-being separately.

Additionally, a questionnaire specially designed for this study with a total of 30 questions and a 4-step ordinal scale for the answers to assess the rating of the participants' subjective structuredness in their everyday life will be filled out. This questionnaire includes, for example, questions on the subjective assessment of whether activities such as eating, sleeping and sport are carried out regularly, whether relapses are associated with irregular daily routines, and whether the craving for alcohol usually occurs at the same time of day. To give the subjects of the control group no indication of the actual aim of the study, the questions that aim at the circadian daily structure are mixed with neutral questions.

Drinking behavior and daily craving will be assessed with questionnaires and the diary, respectively (Table 1). General drinking habits of the last few weeks are surveyed using the AUDIT questionnaire. In addition, the European Addiction Severity Index (EuropASI) is used, which covers not only alcohol and drug consumption but also physical, psychological, financial, and social problem areas of addicted patients. The number of alcoholic drinks and the number of alcohol-free days within the past 30 days is assessed by means of the Timeline Followback 30 (TLFB-30). The diary, which the subjects fill out to collect the Routine Variability Scores, additionally records the highest craving for alcohol on a scale of 0–10 of each day.

According to our secondary hypothesis that the stabilization of circadian rhythms contributes to the reduction of depressive symptoms and increased well-being, changes in the affective state, with a focus on depressive symptoms, are measured with the external assessment questionnaire Hamilton Depression Scale (HAMD) and with the self-reporting questionnaire Inventory of Depressive Symptomatology (IDS-SR). Sleep quantity and quality will be assessed by Pittsburgh Sleep Quality Index (PSQI). Furthermore, for measuring if the DAILY structure program will influence the feeling of self-efficacy, we will employ the Generalized Self-Efficacy Scale (GSES).

As exploratory outcomes, it will be documented how many subjects in the DAILY intervention group and the control groups discontinue the therapy. Furthermore, possible changes of the chronotypes in the course of the study will be defined by the Munich ChronoType Questionnaire (MCTQ).

As alcohol has various negative effects on different blood cells and their functions, we will also take blood samples and assess liver and blood parameters (see Table 2).

**Table 2:** Overview of blood parameters that will be assessed at baseline and at 6-weeks follow-up.

**Liver values**		**Blood cells and other AUD related values**	
**Abbreviation**	**Full name**	**Abbreviation**	**Full name**
GOT	Glutamic Oxaloacetic Transaminase	MCV	Mean Corpuscular Volume
GPT	Glutamate-Pyruvate-Transaminase	Hb	Hemoglobin
GGT	Gamma GT	Vit B12	Vitamin B12
aP	Alkaline Phosphatase	CDT	Carbohydrate-Deficient Transferrin

## Data Management

During the study, all data assessed will be electronically saved. The saved data will be securely stored by password and pseudo-randomization. All data will be subjected to medical confidentiality and general data protection regulations.

## Data Analysis

All statistical analyses will be performed using SPSS software version 25 (SPSS INC., Chicago, IL, USA). Sociodemographic data, chronotype, diary data and blood parameters will be reported in form of descriptive statistics. Continuous variables will be presented as mean ± *SD* and normality of continuous data will be measured by using the Kolmogorov-Smirnov-Test. Accordingly, we will use parametric and non-parametric analyses. For all calculations, the significance level will be determined at α < 0.05.

Data analysis will be performed according to intention-to-treat analysis (ITT) rules. In particular, this means that all subjects who completed the questionnaires of the first session and the 1st week of the diaries are included in the analysis, regardless of whether their subsequent data sets are complete or whether they have dropped out of the study. This is done by imputing missing data with the help of all available data that was assessed until then. Participants who withdraw their consent and were not assessed with baseline measurements will be treated as dropouts. All other subjects regardless from the study condition will be analyzed according to ITT.

The data will be evaluated in three different ways: First, outcomes of the endpoint of the study will be compared between the intervention and control groups using unpaired group analyses (e.g., Student's *t*-test, Mann-Whitney *U*-test, Kruskal-Wallis test, one-way ANOVA). Secondly, within-subject analyses of the intervention and control groups are performed, comparing the changes between the start and end of the study of the two groups (e.g., two-way ANOVA). Thirdly, the data are calculated independently of group membership in two-dimensional linear regression using the Routine Variability Scores as an independent variant.

Additionally, statistical differences in the frequency of dropouts between the two groups are calculated using chi-square tests and survival analyses. Multiple regression analyses will be applied to investigate the degree to which predictor variables (i.e., Routine Variability Scores, depression level, severity of alcohol consumption behavior, and self-efficacy score) contribute to outcomes of primary and secondary measures.

## Discussion

The DAILY intervention is a new concomitant treatment approach for patients suffering from AUD, which is based on the strengthening of the patients' circadian system with the targeted use of *Zeitgebers* such as sleep, meal, and activity times. The timing for the regular use of the *Zeitgebers* is personalized and tailored to the individual patients including both their genetically imprinted circadian characteristics as well as individual social obligations, such as working hours. Hence, the personalized design should therefore make the strengthening of physiological rhythms as effective as possible by taking individual characteristics into account on the one hand, and on the other hand as easy to implement as possible by including environmental constraints. Each individual is determined by a genetic constellation that defines optimal times for sleeping, eating and physical exertion (e.g., sports). In turn, these specific actions serve as *Zeitgebers* for the circadian system. Consequently, the circadian clock and the *Zeitgebers* form a positive feedback system, which leads to mutual reinforcement. Thus, this bidirectional influence can be used to strengthen and stabilize circadian clocks. Our therapy makes sure that actions serving as *Zeitgebers* are performed at the same time on all days. This preserves the circadian system from repeated shifts and ensures that a fixed phase relationship among physiological processes, and thereby endogenous synchronization, can be established. However, there are many individuals who are subject to social times that they cannot completely elude in everyday life and which may change between work and work-free days. The primary focus of our study is to test whether AUD patients benefit from increased regularity of *Zeitgeber* exposure which requires adherence to the same schedule on workdays and workfree days. Thus, our therapy bears the risk of a permanent misalignment or sleep deprivation for some participants. Therefore, if the endogenous rhythms of the test subjects, who are not in the position to choose their sleeping times on most days of the week, deviate more than 2 h from constrained social times, they are advised to adjust their biological time using light therapy to avoid chronic misalignment with the environment. Thus, our therapy ensures endogenous alignment of rhythms and additionally counteracts the so-called social jetlag, which is also associated with increased alcohol consumption (68, 69). However, if internal and external rhythms are found to be extremely different, subjects may not be suitable for this study.

As described above, the brain's reward system and the development and maintenance of substance use disorders are particularly closely related to the circadian clock. These mechanistic explanations will also be discussed with the participants of DAILY. We believe that psychoeducation offers the patients the opportunity to learn about tangible reasons for the importance of regular daily structures, increases their awareness of the timing of their daily activities and enhances the general compliance during therapy. Therefore, DAILY consists of a unique and balanced concept that builds on three main components:

1. psychoeducation about sleep hygiene and the circadian system, its connection to AUD, and the physiological mechanisms through which a regular daily structure may improve therapeutic success,
2. strengthening of the circadian system and thereby optimizing physiological processes and neuronal communication,
3. additional positive psychological effects through the feeling of stability, control, and self-management in the often-difficult transition period between inpatient and outpatient care.

In the past, different chronotherapeutic treatments with different therapy aims have been described and clinically tested in mood disorders, but, to our knowledge, never in addiction disorders. Furthermore, in our opinion, personalization, which aims at balancing genetically and socially determined rhythms, has not been sufficiently incorporated in previous chronotherapeutic approaches. For example, the most common chrono-treatment is light therapy, which has two goals, increasing the daily light dose and shifting the chronotype from late to early. Light therapy is widely applied and highly effective as it can reach similar positive effects on mood as antidepressant drugs (70). Light therapy is known to be effective for the treatment of seasonal affective disorder, MDD and was shown to be effective in bipolar disorder patients (71, 72). Similarly, sleep advance therapies are used to shift late chronotypes to earlier hours, which has been shown to have positive effects on mood. The effect, however, is primarily related to the patient's mood and is not of long duration. The therapeutic effects of total or partial sleep deprivation on mood are even shorter and a connection with the circadian system is not proven (73). These approaches have the only aim of adapting the chronotype to environmental rhythms and often follow uniform protocols. However, trying to adapt patients to the same early chronotype can be very difficult for those subjects whose endogenous chronotype differs widely.

Contrarily, already in the late 90's, an approach that considered individual circadian traits, the so-called interpersonal social rhythm therapy (IPSRT), has been tested successfully in bipolar patients (60, 74), but, to our knowledge, never for AUD patients. IPSRT focuses primarily on stabilizing social rhythms such as eating regularly with other people and thus improving social interactions and relationships (75). Its efficacy has been proven multiple times (61, 76). Interestingly, there is a renewing increase in awareness and clinical interest for therapy approaches taking the effects of regular daily structures and sleep hygiene into account (19, 63, 77). In accordance with IPSRT, we believe that a more ideal form of chronotherapy is not necessarily the attempt to change endogenous circadian rhythms (e.g., chronotypes), but to exploit and strengthen them as much as possible in order to achieve greater robustness of the individual circadian clock.

Cognitive behavioral therapy for insomnia (CBT-I) focuses, like parts of DAILY, on a regular sleep schedule, stimulus control, and improved sleep hygiene. Other core components of CBT-I are sleep restriction and relaxation techniques, which are not used in DAILY. CBT-I treatment in AUD patients effectively reduces insomnia, associated negative cognitions, and improves sleep hygiene, but, however, is not successful in changing drinking behavior (78, 79). Apart from sleep problems, AUD patients often suffer from irregular routines throughout the day, including the timing of meals. Thus, in contrast to the focus of CBT-I on sleep improvement, DAILY aims to stabilize the circadian system of the entire body, including clocks in peripheral organs and reward-regulating brain areas, which in our opinion is more likely to improve alcohol outcomes.

In summary, in DAILY we aim to increase stability and regularity of circadian rhythms of AUD patients by creating daily routine schedules which will consider as many endogenous circadian preferences as possible, but also social obligations. Actions whose timing is determined by social constraints will be integrated in the daily routine schedules to make their execution feasible, and their timing is kept the same on all days to create endogenous synchronization. In subjects whose daily routine schedule deviates more than 2 h from endogenous rhythms, light therapy will be used to adjust their chronotype to avoid social jetlag. In this way we obtain a high degree of personalization and can try to create the best possible daily profile for each subject.

### Limitations

At this state, however, DAILY has some limitations as it is designed as a pilot study and so, it aims primarily at proofing the principle study concept and the overall impact of a daily structure therapy in AUD patients. A limitation of the current design is that it does not include objective physiological and behavioral measures of circadian rhythm improvement such as (e.g., melatonin rhythms or actigraphy, but only focuses on more subjective behavioral rhythms based on diary entries). However, the primary aim of this pilot study is the proof whether a therapeutic approach to improve sleep hygiene and regularity in daily routine can generally improve AUD symptoms.

Furthermore, inpatients and semi-inpatients are included into our study. Inpatients and semi-inpatients usually already have a more structured daily routine than outpatients since they are given a regulated daily structure by the clinical routines and the fixed therapy hours. Therefore, the determination of individual circadian preferences and characteristics might be limited. However, in our clinic, where the study is currently taking place, patients have no mandatory eating or sleeping hours and can leave the ward and day clinic outside therapy hours. Thus, the test persons can in principle follow their own rhythms, which is often taken advantage of, especially for food and snacks in the surrounding areas. Nevertheless, many test persons report that they generally have more structured daily routines in the clinic than at home, as they adapt to the rhythms of their fellow patients as well as the rhythms of the clinic meals offered.

Furthermore, there are also test persons who, after detoxification or after their stay in the day clinic, go for further treatment to other clinics where they are integrated into a daily structure. Since this daily structure does not necessarily correspond to the subject's circadian characteristics, the analysis of pilot data must consider that these test persons cannot always independently follow the daily schedule during the study. Accordingly, it is possible that the implementation of the DAILY program is more suitable for clinics from which patients are usually discharged home.

Additionally, inpatients and semi-inpatients often have a more severe symptomatology than outpatients. Thus, results of this pilot study may not be completely representative for all patients suffering from AUD. However, it might be possible, that the therapeutic effects of the DAILY treatment will be even more pronounced in outpatients. AUD outpatients often suffer from less severe symptoms and might particularly benefit from a daily structure therapy as they are usually at higher risk to have a more disorganized and unstructured daily life. However, this is not tested in the current study. Generally, it is expected that DAILY therapy will be particularly suitable for patients who have severe irregularities in their everyday life. AUD patients, however, who have a very regular daily routine of their own accord, will probably not benefit to the same extent from the therapy presented here.

Another limitation of the current study design is that no long-term follow-up is scheduled. However, this limitation will be addressed in a future study with a more longitudinal perspective.

### Outlook

In future studies, the determination of individual circadian traits will be optimized in two approaches. First, outpatients will be included because these patients most likely organize their day following primarily their endogenous clock compared to inpatients who are integrated into the daily structures of the clinic. Second, we will also use actigraphy on the one hand to receive a more objective measure of regularity of rest-activity rhythms and on the other hand because filling out diaries carries a high risk of non-compliance and therefore a high risk of data gaps.

In recent years, great progress has been made in the development of machine learning methods for the evaluation of circadian variables of actimeter data, with which for example the circadian phase can be determined (80–83). In this regard, a newly developed DLMO prediction model based on actimetry data, which can be used to quantify circadian misalignment, seems particularly useful to quantify irregularities in circadian rhythms and behavior (84). Therefore, the application of actimeters and the use of mathematical algorithms for analysis will be part of future and more comprehensive studies. The results obtained can in turn be used to adjust and refine daily structure plans of the individual participants of our second study cohort.

Beyond that, we will try to identify times of the day when alcohol cravings are particularly high. Knowing about these times, we will encourage the patients to use these hours to have recourse on learned skills (doing sports, contacting close persons etc.) in order to overcome this period more successfully.

The overall goal of this and more extended future studies is the development of an accompanying therapy for AUD patients that is uncomplicated in its use and easy to implement in the clinical routine. This therapy should enable AUD patients to bring more regularity and structure into their everyday life and thus to strengthen their circadian system. This, in turn, may be a critical component in overcoming alcohol withdrawals and in preventing relapses more successfully.

## Ethics Statement

The studies involving human participants were reviewed and approved by Local Ethics Committee of the Ludwig Maximilian University Munich. The patients/participants provided their written informed consent to participate in this study.

## Author Contributions

AH and DL wrote the manuscript. All authors were involved in the design, conceptualization of the study, editing the manuscript, and approving the final version.

## Conflict of Interest

The authors declare that the research was conducted in the absence of any commercial or financial relationships that could be construed as a potential conflict of interest.
