# Supplementary material for: DAILY—A Personalized Circadian Zeitgeber Therapy as an Adjunctive Treatment for Alcohol Use Disorder Patients: Study Protocol for a Randomized Controlled Trial
Source: Front Psychiatry. 2021 Jan 14;11:569864. doi: 10.3389/fpsyt.2020.569864 (PMC7840704; doi:10.3389/fpsyt.2020.569864)
Supplement: Supplementary file 1 [file Table_1.pdf]

Diary

Week __

		Day 1	Day 2	Day 3	Day 4	Day 5	Day 6	Day 7
	Today´s date							
Morning	Time of waking up							
	Time of getting up							
	Alarm clock (yes/no)							
	Sleep quality (min **0** - max **10**)							
	Working day (yes/no)							
Evening	Time of going to bed							
	Time of lights off							
	Minutes between lights off and sleep							
	Today´s mood (min **0** - max **10**)							
	Time of max. craving							
	Intensity of max. craving (min **0** - max **10**)							
	00:00							
	00:30							
	01:00							
	01:30							
	02:00							
	02:30							
	03:00							
	03:30							
	04:00							
	04:30							
	05:00							
	05:30							
	06:00							
	06:30							
	07:00							
	07:30							
	08:00							
	08:30							
	09:00							
	09:30							
	10:00							
	10:30							
	11:00							
	11:30							
	12:00							
	12:30							
	13:00							
	13:30							
	14:00							
	14:30							
	15:00							
	15:30							
	16:00							
	16:30							
	17:00							
	17:30							
	18:00							
	18:30							
	19:00							
	19:30							
	20:00							
	20:30							
	21:00							
	21:30							
	22:00							
	22:30							
	23:00							
	23:30							
	Comments:							

To fill in:

"X" = Main meal

"Sn" = Snack

"Dc" = Drink containing caffeine

"Ds" = Drink containing sugar or fruit juice

"Mi" = Milk or dairy product

"Sp" = Sports

"Na" = Nap

Table S1: Diary for recording sleeping, eating, and craving times. In the upper part of the diary, data on sleep times and sleep quality are collected. Sleep times are divided into the times of going to bed or getting up and the times of actual falling asleep or waking up, as the latter allow more reliable conclusions to be drawn about endogenous circadian rhythms of the test persons. It also notes whether the current day is a working day or a non-working day and whether an alarm clock was used to wake up. Furthermore, the average mood state, the highest intensity of craving for alcohol, and the time of the strongest craving of each day are recorded. In the lower part of the diary, all meals are entered in a table with a resolution of 30 min. When entering meals, a rough distinction is made between different foods and the size of the meal. Times of possible sports activities and naps are also entered in the table. At the bottom of each day there is space for possible comments, e.g. special events of the day which might have influenced the daily structure. The diary is a modification of an unpublished template, which we have been kindly provided by the laboratory of Prof. Till Roenneberg of the Institute of Medical Psychology at the Ludwig Maximilian University of Munich.
